# To Eat or Not to Eat? Debris Selectivity by Marine Turtles

**DOI:** 10.1371/journal.pone.0040884

**Published:** 2012-07-19

**Authors:** Qamar Schuyler, Britta Denise Hardesty, Chris Wilcox, Kathy Townsend

**Affiliations:** 1 School of Biological Sciences, Moreton Bay Research Station, University of Queensland, Dunwich, Queensland, Australia; 2 Ecosystem Sciences, Commonwealth Scientific and Industrial Research Organisation, Atherton, Queensland, Australia; 3 Marine and Atmospheric Research, Commonwealth Scientific and Industrial Research Organisation, Hobart, Tasmania, Australia; University of Wales Swansea, United Kingdom

## Abstract

Marine debris is a growing problem for wildlife, and has been documented to affect more than 267 species worldwide. We investigated the prevalence of marine debris ingestion in 115 sea turtles stranded in Queensland between 2006–2011, and assessed how the ingestion rates differ between species (*Eretmochelys imbricata* vs. *Chelonia mydas*) and by turtle size class (smaller oceanic feeders vs. larger benthic feeders). Concurrently, we conducted 25 beach surveys to estimate the composition of the debris present in the marine environment. Based on this proxy measurement of debris availability, we modeled turtles’ debris preferences (color and type) using a resource selection function, a method traditionally used for habitat and food selection. We found no significant difference in the overall probability of ingesting debris between the two species studied, both of which have similar life histories. Curved carapace length, however, was inversely correlated with the probability of ingesting debris; 54.5% of pelagic sized turtles had ingested debris, whereas only 25% of benthic feeding turtles were found with debris in their gastrointestinal system. Benthic and pelagic sized turtles also exhibited different selectivity ratios for debris ingestion. Benthic phase turtles had a strong selectivity for soft, clear plastic, lending support to the hypothesis that sea turtles ingest debris because it resembles natural prey items such as jellyfish. Pelagic turtles were much less selective in their feeding, though they showed a trend towards selectivity for rubber items such as balloons. Most ingested items were plastic and were positively buoyant. This study highlights the need to address increasing amounts of plastic in the marine environment, and provides evidence for the disproportionate ingestion of balloons by marine turtles.

## Introduction

### Marine Debris and Sea Turtles

Marine debris has become a significant global issue in recent years. Over the past five decades, global plastic production has increased exponentially [1]. Concurrently, plastic has rapidly become the dominant component of marine debris, representing as much as 80% in areas [2], [3]. Despite increasing awareness of the prevalence of plastic debris, there is little data on the total amount of debris in the marine environment, or how that quantity may have changed through time [4], [5]. The impacts of this debris, however, have been widely documented, with at least 267 marine species known to be affected by anthropogenic debris [6]. Debris can cause a number of different problems for wildlife, but all fall under two main categories: impacts from entanglement and from ingestion. Entanglement can kill wildlife by drowning or inhibiting the ability to escape predation or feed normally, while the implications of debris ingestion include death through perforation or impaction of the digestive system [7]. Additional sublethal impacts include dietary dilution [8] and exposure to chemicals leaching from plastic [9]. All six species of sea turtle listed on the IUCN Red list [10] have been documented to ingest debris [6].

Globally, estimates of debris ingestion rates in turtles vary dramatically with geographical region, species, and year. Recent work from South American populations of marine turtles found that up to 100% of stranded turtles contained marine debris in their gastrointestinal systems [11]. The problem affects turtles of all life stages, from post-hatchlings through adults [12]–[14]. It is unknown why sea turtles ingest plastic: one hypothesis is that plastic bags resemble a typical prey item, jellyfish [15]. Although this may be the case for turtles that ingest plastic bags, it does not explain the ingestion of other forms of plastic, Styrofoam, rubber, ropes, and the myriad of other items that have been found in turtles [16]–[21]. Although sea turtles can and do utilize olfaction to orient to prey, they are primarily visual feeders [22]. The presence of at least three different cone photopigments in sea turtle retinas, as well as electrophysiological measurements and behavioral studies, indicate their ability to discriminate color [23]–[25]. This color vision may play a role in feeding choices, as has been demonstrated in laboratory trials [25]–[28]. If this is the case, monitoring the color of debris ingested by turtles may offer insights to the reasons why turtles eat debris, and may also lead to conservation and management recommendations. Color preference (or avoidance) has already been investigated as a possible method for decreasing sea turtles’ interactions with the bait used in longline fisheries. Unfortunately, although turtles exhibit a preference for natural bait over blue dyed bait in a laboratory situation, dyed bait does not appear to reduce long line interactions in field trials [28].

### Hawksbill Turtle and Green Turtle Life History

Both *Chelonia mydas* (green turtles) and *Eretmochelys imbricata* (hawksbill turtles) begin their developmental phase in the open ocean before recruiting back as latter stage juveniles to the coastal environment, where they spend the rest of their lives [29]. Before recruitment, the post-hatchling turtles and early stage juveniles live and feed primarily at the ocean’s surface, occasionally diving to shallow depths [30]. They are thought to drift with the currents, aggregating in downwelling lines along with other floating biological material and debris [12]. During this phase they feed on plankton, comprising primarily molluscs, crustaceans, and gelatinous organisms [20]. Living in downwelling zones may provide the young turtles with increased shelter and food opportunities, but also exposes them to concentrated areas of floating debris.

The turtles’ feeding behavior changes dramatically once they recruit to the nearshore environment. The size of first recruitment varies between species and geographic region, but on the east coast of Australia, green turtles recruit at approximately 40 cm curved carapace length (CCL) [31], and hawksbill turtles at >35 cm CCL [32]. These coastal turtles feed primarily on benthic resources such as seagrass, crustaceans, sponges, and algae, although even primarily herbivorous green turtles will opportunistically feed on jellyfish when available [33], [34]. Green turtles are known to be selective in their feeding, choosing particular species of seagrass over others, and even tending “grazing plots” to gather new shoots that are easier to digest and have higher nutritional value [35]. Hawksbill turtles also feed selectively, preferentially ingesting certain items even when they are less readily available in the environment [36].

With this diversity in feeding habitat and style between pelagic and benthic stage turtles, we predict that exposure to marine debris would differ between the two groups. These differences could be exacerbated by the variability in types, colors, and quantities of debris present in benthic and oceanic environments [1]. It is likely that pelagic stage turtles, which drift in current lines along with other floating debris, would be at greater risk of marine debris ingestion than the larger benthic animals [12], [33]. Because of their different diets and feeding styles, pelagic and benthic turtles may vary not only in the amounts of debris they ingest, but also in the type. Analyzing the type and color of debris gives us metrics to compare the variability in debris selection between turtles at different life stages.

Our aims were to 1) investigate whether the incidence of debris ingestion varies between turtle species and between life history stages, 2) determine whether turtles preferentially ingest particular types and colors of debris by comparing the ingested debris to what is available in the environment, and 3) analyze whether selectivity varies between life history stages and between species.

## Materials and Methods

This research was reviewed and approved by the University of Queensland Native/Exotic Wildlife and Marine Animals (NEWMA) Animal Ethics Committee. The ethics approval number is ANRFA/MBRS/182/11. Animals involved in the study were already deceased, so no steps were taken to ameliorate suffering.

**Table 1:** Debris ingestion probability for pelagic and benthic stage turtles, and characteristics of these turtles.

	Total number of turtles	Number of turtles having ingested debris (% of total)	Range of CCL (cm)	Mean CCL (+/− s.e.)
All turtles	115	39 (33.9%)	5.4–105.8	39.08±19.35
Pelagic	22	12 (54.5%)	5.4–34.9	20.44±11.61
Benthic	93	27 (29.0%)	35.31–44.7	47.37±16.08

From 2005–2011, 115 turtles were obtained in southeast Queensland from two sources: dead stranded sea turtles from North Stradbroke Island (n = 64), and sea turtles that did not survive treatment at the marine wildlife rehabilitation facility at Underwater World, in Mooloolaba (n = 51). Eighty-eight were green sea turtles (*C. mydas*), 24 were hawksbill turtles (*E. imbricata*), 2 were loggerhead turtles (*C. caretta*) and one was a flatback turtle (*N. depressus*). The turtles ranged from 5.4–105.8 cm CCL, with a median size of 43.4 cm. Because of the small sample size of loggerhead and flatback turtles, all investigations of inter-species differences were restricted to green and hawksbill turtles.

**Table 2:** Characteristics of debris items found within turtles that had ingested debris, for which detailed debris information is available (n = 33).

	Number of items ingestedper turtle (avg ± s.e.)	Weight of items ingested(avg ± s.e.)	% of positively buoyant items ingested (avg ± s.e.)
Turtles (n = 33)	1–329 (31.7±10.18)	n.d.–10.41 g (1.58±0.50)	81.59±7.09
Pelagic (n = 11)	1–69 (22.5±6.78)	0.03–2.16 g (0.86±0.33)	80.51±13.91
Benthic (n = 22)	1–329 (38.8±15.01)	n.d.–10.41 g (1.89±0.70)	81.99±8.47

**Table 3:** Number of turtles ingesting each type of debris, and proportions of total for different debris categories (out of n = 33 turtles for which detailed debris categories are available).

Type of debris	Number of turtles (and % of total) withingested debris	Percentage of total amount of debrisingested by all turtles (n = 1057)
Hard plastic	19 (57.6%)	33.11
Soft plastic	24 (72.7%)	34.25
Plastic rope/string/twine	14 (42.4%)	13.06
Non plastic rope	1 (3.0%)	1.80
Packing straps	1 (3.0%)	3.12
Fishing items	15 (45.5%)	4.73
Balloons	10 (30.3%)	3.20
Other rubber	5 (15.2%)	0.9
Foam	4 (12.1%)	3.50
Other	10 (30.3%)	2.33

Necropsies were performed on all animals using standard techniques [37]. Contents of the gastrointestinal system were sieved to retrieve any foreign matter. Debris found in the turtles was washed and stored for analysis. Each piece of debris was weighed (to within 0.01 g) and categorized into one of six main categories and additional subcategories, based on a classification system combining both composition and morphology. The categories were: hard plastic, soft plastic, foam, rope/string, rubber, and miscellaneous (includes glass, metal, paper, cloth). Hard and soft plastic objects were further categorized by color. Positive or negative buoyancy was also measured for each item. For six of the turtles, debris samples were not retained; so detailed categorical information is not available. The majority of rope and string items (>85%) were composed of plastic material, but were categorized separately due to their morphology.

**Figure 1:**
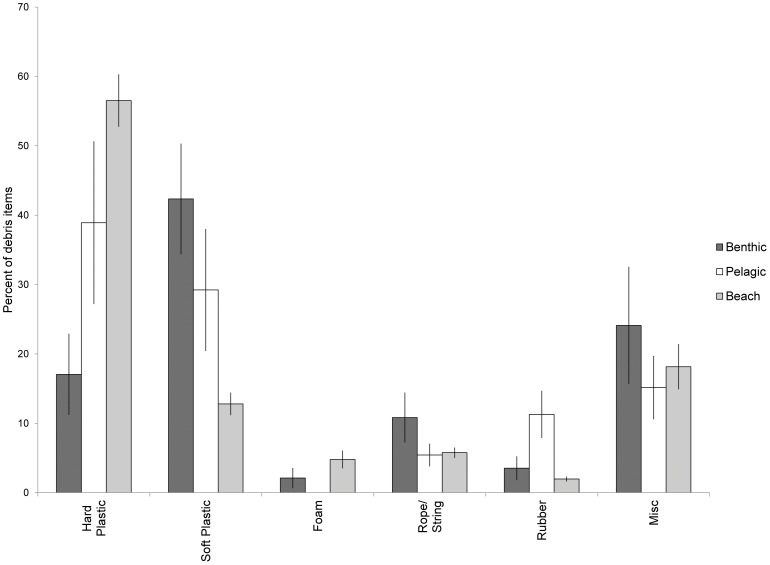
Debris types found in turtles and on beaches. Types of debris found during beach surveys, and in the gastrointestinal system of stranded sea turtles. Reported as an average of the percentage of each category found within each animal (benthic n = 22, pelagic n = 11), and during each beach survey (n = 25). Error bars indicate standard error.

We calculated the frequency of ingestion of each category of debris using the following equation:
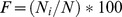
where 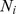 is the number of turtles having ingested a particular type of debris, *i,* and *N* is the total number of turtles for which detailed debris information is available [38]. We also determined the relative percent abundance of debris types ingested by each turtle by calculating the percentage



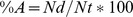
where *Nd* is the number of items of each type of debris, and *Nt* is the total number of items of debris found in the turtle. Turtles were divided into two groups; pelagic sized feeders and benthic sized animals. We categorized pelagic feeders as those animals smaller than 35.0 cm, and benthic feeders as those >35 cm CCL.

To estimate availability of anthropogenic debris, we conducted beach surveys between 2009–2011 on four beaches on N. Stradbroke Island and four beaches on the Sunshine coast, in the region where the Underwater World turtles stranded. We collected all pieces of debris over 5 mm found in a 100 m long strip transect running parallel to the water line on each beach. The strip transect width varied depending on tide and the beach in question, but encompassed the distance from the waterline to the dominant landward vegetation line. Beach debris was assigned to the same categories as debris found in turtles. We calculated the relative abundance of each type and color of plastic debris found in the environment using equations parallel to those above. For simplicity of analysis, and because no individual color represented more than 10% of the sample, we combined our color and debris types to create 10 categories in order to measure selectivity indices for the turtles. These types were: hard white plastic, hard colored plastic, hard clear plastic, soft white plastic, soft colored plastic, soft clear plastic, rope/string, rubber, foam, and miscellaneous.

**Figure 2:**
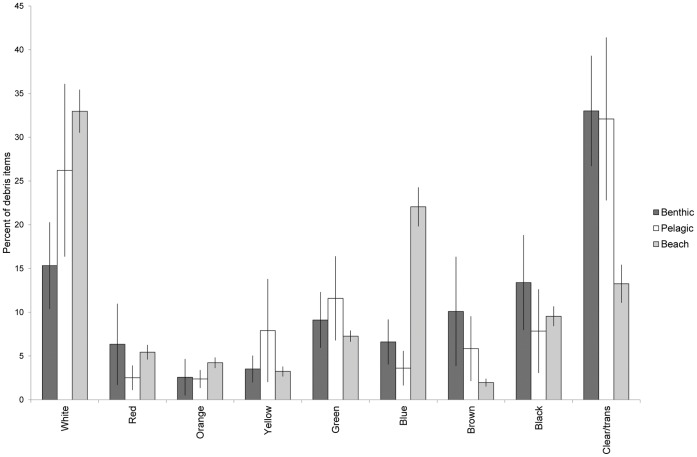
Debris colors found in turtles and on beaches. Colors of debris found during beach surveys, and in the gastrointestinal system of stranded sea turtles. Reported as an average of the percentage of each category found within each animal (benthic n = 22, pelagic n = 11), and during each beach survey (n = 25). Error bars indicate standard error.

**Figure 3:**
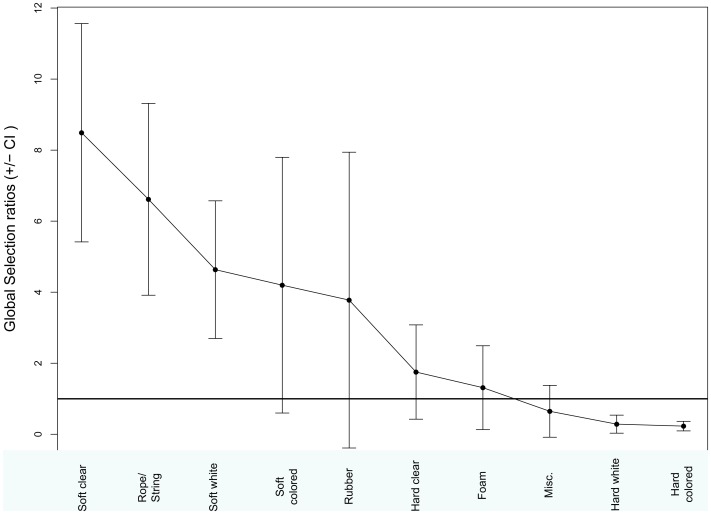
“Jellyfish” balloon. Beach-washed balloon found after brittle fracture. Note the resemblance to jellyfish, common prey items for turtles.

**Figure 4:**
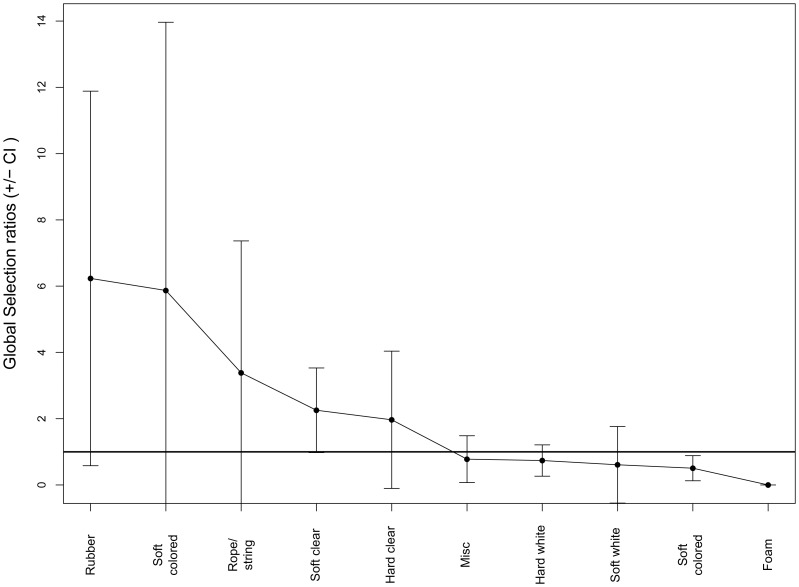
Manly selectivity measure for benthic turtles. Selectivity index for various types of debris ingested by benthic-feeding turtles. Where index is larger than one, selectivity for that item is greater than its availability in the environment. Error bars indicate 95% confidence interval.

We used a binomial regression to predict the probability of ingestion based on the descriptive variables CCL and species (*C. mydas* and *E. imbricata*), and a chi square analysis to determine differences between ingestion probabilities for life history stages. For the turtles that had ingested debris, we tested the relationship between CCL and debris load (both total weight and also number of pieces of debris ingested) using a generalized linear model (GLM, Gaussian model). Finally, we calculated Manly’s selectivity ratio for each debris category ingested for both life history stages. This technique has been widely used to estimate resource selection functions for habitat or diet [39]. The index takes into account the availability of each type of resource in the environment. A value greater than 1 indicates a positive selectivity for that category, while a value less than one suggests that turtles avoid ingesting that type of debris compared to what is available in the environment. All analyses were performed using R version 2.14, package nnet and adehabitat [40]–[42].

**Figure 5:**
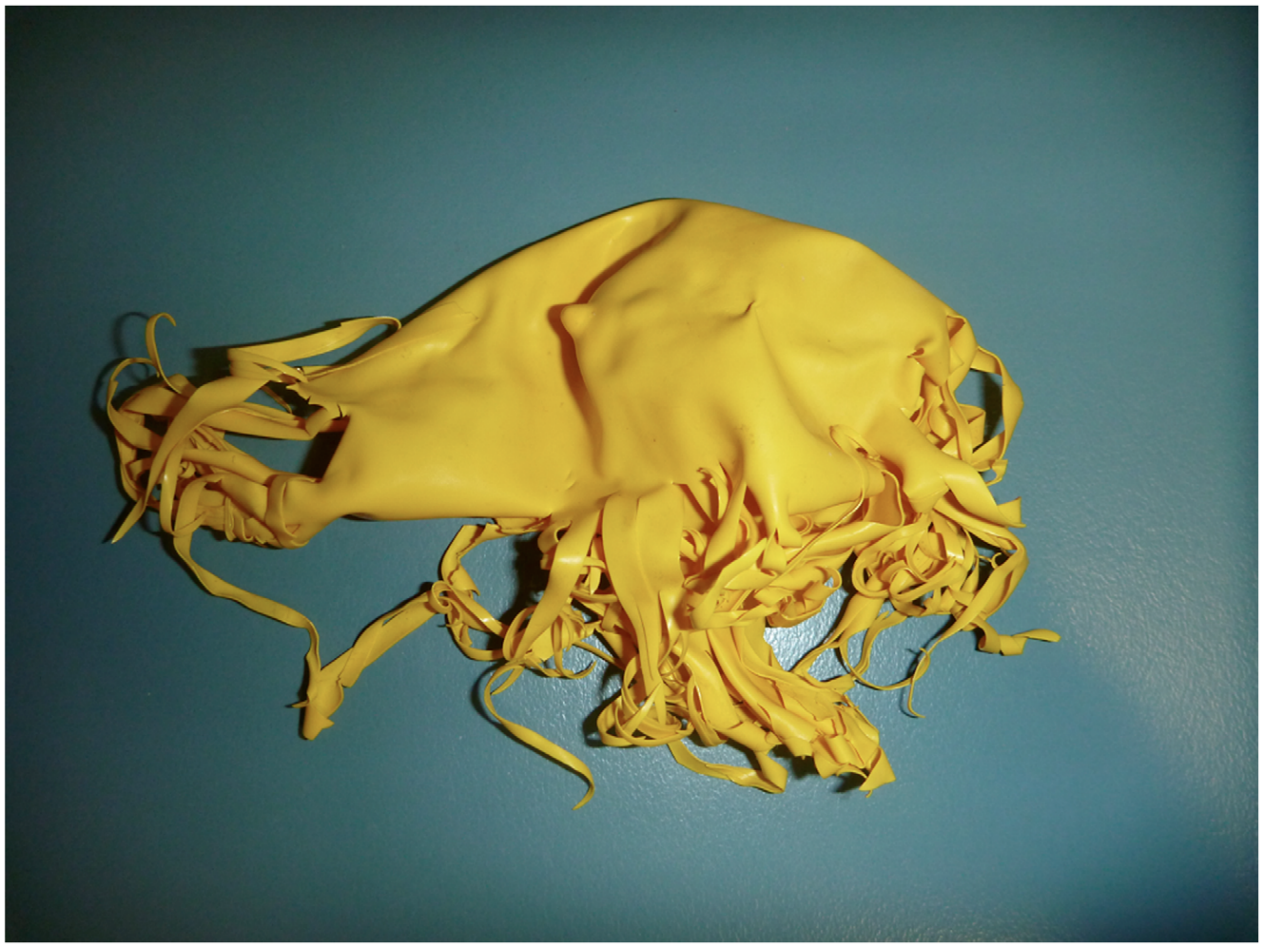
Manly selectivity measure for pelagic turtles. Selectivity index for various types of debris ingested by pelagic-feeding turtles. Where index is larger than one, selectivity for that item is greater than its availability in the environment. Error bars indicate 95% confidence interval.

## Results

### Debris Ingestion

Of the 115 necropsied animals, 22 were oceanic-size turtles, and 93 were from benthic habitats (Table 1). A total of 33.9% (N = 39) of the turtles were found to have ingested debris. Ingestion frequencies differed significantly between oceanic (n = 12, 54.5%) and benthic-sized turtles (n = 27, 29.0%), despite our uneven sample sizes (chi-square 4.09, df = 1, p = 0.043). There was a significant negative correlation between CCL and probability of debris ingestion (p = 0.0338), but no correlation with the weight of debris ingested (p = 0.942), or total number of pieces of debris ingested (p = 0.215). Nor was there a significant effect of species on the probability of ingesting debris (p = 0.445), or a species by size interaction (p = 0.430). Because we do not have detailed debris information for six of the turtles, calculations on the weight and total number of debris items were carried out only on n = 33 turtles.

A total of 1057 pieces of debris were ingested by 33 turtles. The number of pieces ingested by each individual turtle ranged from 1–329 with an average of 31.7±10.18 (s.e.) pieces per turtle. The total weight of all items found within each turtle ranged from non-detectable (<0.01 g) to 10.41 g. The average proportion of positively buoyant items ingested by the turtles was approximately 80% and did not vary significantly between the two life stages (Table 2). Hard plastic comprised 33.11% of the total number of debris items ingested, 34.25% was soft plastic, and plastic rope followed at 13.06% (Table 3). Including fishing line and packing straps, the total amount of plastic debris ingested by turtles made up nearly 90% of all debris items. When data were analyzed by life history stages, oceanic sized turtles ingested significantly more hard plastic and rubber than benthic turtles, while benthic turtles ingested more foam and rope than pelagic turtles (Fig. 1). Colors varied between the two classes, but not significantly. The color of plastic debris found in both pelagic and benthic turtles was primarily clear or translucent, followed by white (Fig. 2). Black debris comprised mainly black plastic bags, while green and blue were mostly plastic rope and string. Other colors (red, orange, yellow, and brown) were found in very small quantities.

### Environment and Selectivity

The majority of the debris found during all beach surveys was hard plastic, with only one other category (miscellaneous) at over 15% (Fig. 1). White debris made up over 30% of collected items, followed by blue and clear/translucent (Fig. 2). Using beach debris as a measure of environmental availability, Manly’s selectivity ratio highlighted the selectivity differences between turtles from different life stages. Benthic sized turtles showed strong selectivity for soft plastics in general, particularly for clear soft plastics, and for rope. They appeared to avoid hard white and colored debris (Fig. 3). Pelagic turtles had the highest selection ratios for rubber, rope, and hard plastic, but these did not differ significantly from the environment (Fig. 4).

## Discussion

Marine debris or more specifically, plastic ingestion by sea turtles is a global phenomenon, affecting populations worldwide. The vast majority (nearly 90%) of all ingested items in this study were plastic in origin, a finding common to most other studies reporting debris ingestion in turtles [16], [43]–[45]. This reflects the significant contribution of plastic to the global marine debris problem [5].

This study discovered no significant differences in debris ingestion between the species investigated; *C. mydas* and *E. imbricata*. This is perhaps due to the fact that the two species exhibit similar feeding behavior, with smaller turtles feeding pelagically, and larger turtles shifting to benthic feeding [46]. Although species had little effect on debris ingestion rates, size did. The probability of debris ingestion was inversely correlated with size (CCL), and when broken down into size classes, smaller pelagic turtles were significantly more likely to ingest debris than larger benthic feeding turtles. These results are in line with research conducted by Balazs [47] and Plotkin and Amos [17], though other studies found no significant relationship between size or life history stage and debris ingestion. Most of these studies investigated the relationship between turtle size (CCL) and weight, number, or size of the pieces of debris ingested, but did not analyze the probability of debris ingestion [16], [44], [45], nor did they investigate differences between life history stages [16], [45]. Bjorndal’s [13] analysis of ingestion probability and size class of green turtles suggested that a higher percentage of turtles <30 cm had ingested debris in comparison to their larger counterparts, however this difference was not significant. Size class or life history stage appears to be an important factor in determining the probability of debris ingestion, but the number of pieces, total weight, or volume of ingested debris rarely correlates with size class or life history stage, as highlighted by this and other studies.

Turtles in this study from different life history stages varied not only in their likelihood of ingesting debris, but also in the types of debris ingested. Pelagic turtles ingested significantly more rubber and hard plastic than did benthic feeding turtles, who primarily ingested soft plastic (Fig. 1). While there was not a significant difference in the colors ingested between the two groups, they did differ from what was available in the environment, ingesting clear debris in greater proportions, and blue at lower proportions (Fig. 2). Manly’s selectivity ratio, and its significance level, also varied with life history stage. Neritic turtles actively selected white and clear soft plastics, while avoiding hard white and colored plastics (Fig. 1). They also showed selectivity for rope and string, but this could be an artifact of the way the samples were tallied. Counts of the total number of items were used to quantify the amount of debris in each category. Multi-stranded rope and string may more readily unravel into smaller (and therefore, more numerous) pieces within the gastrointestinal system than other types of debris, which could be reflected in our results.

Pelagic turtles were much less selective than their neritic counterparts, with most of their selectivity indices not found to be significantly different to environmental levels. Only foam (with zero pieces ingested) and hard colored debris fell significantly below 1, indicating avoidance of these categories. Interestingly, the single highest preference in the pelagic turtles was for rubber. Although the preference was not statistically significant, this may be due in part to the smaller sample size of the pelagic turtles. Of the 41 pieces of rubber found inside all turtles, 32 pieces (78%) were fragments of balloons. When helium balloons are released into the environment, they rise to a height of approximately 8 kilometers before undergoing a process known as “brittle fracture”, where the balloon fragments into long strands [48]. The resulting debris bears a strong resemblance to jellyfish or squid (Fig. 5). Indeed, the brittle fracturing of balloons creates tentacle-like structures typical of *Scyphomedusae* which all species of sea turtles have been documented to eat [46], [49]–[51]. This may be the cause for the high ingestion selectivity seen in both pelagic and neritic turtles. Several studies have reported ingestion of balloons by sea turtles [11], [17], [52], [53], and anecdotal evidence exists for ingestion of balloons by whales and dolphins [54]. Worldwide cleanups sponsored by the Ocean Conservancy over the past 25 years have found over 1.2 million balloons, or about 0.7% of all debris items collected [55]. This is in line with our study, which found a total of 0.9% of rubber items on the beach. Although balloons and other rubber items make up only a small fraction of the total amount of debris collected, the current data indicating that turtles may selectively ingest balloons and other rubber could provide guidance for policy makers addressing mass balloon releases.

The differences in debris preference and selectivity may be a result of feeding styles; young pelagic turtles live an epipelagic lifestyle, floating at the surface and feeding within the top five meters [30]. As they drift with the currents, encountering pelagic gyres and downwelling zones where debris accumulates, they may be susceptible to accidental or purposeful ingestion of debris along with their natural food sources. The presence of encrusting organisms further blurs the line between food and debris. Post hatchlings are thought to be relatively non-selective feeders [56], a finding supported by this research. Conversely, benthic-feeding green turtles and hawksbill turtles are thought to be more selective about their diet [35], [36]. They also may be less likely to come into contact with plastic marine debris, much of which is positively buoyant [57], [58]. However, they also eat gelatinous organisms, which are usually soft and transparent, much like the debris that they most commonly ingest. Our findings lend further support to the hypothesis that turtles mistakenly eat plastic because of its similarity to jellyfish [15]. Other factors may also contribute to the differences in ingestion rates; for example as turtles grow, the internal diameter of their digestive tract becomes larger, making it easier for plastics to pass through, and not accumulate. Pelagic turtles, therefore, may experience a higher risk of mortality from debris ingestion, not only because they are more likely to ingest debris, but also because they are smaller in this life history stage than they are in the benthic stage and their digestive tract is correspondingly smaller. Hence, this may result in an increased possibility of impaction or perforation of the gastrointestinal tract.

There are limitations to using beach surveys as a proxy for the debris that sea turtles encounter. Differences in buoyancy, degradability, and other characteristics may result in certain types of marine debris more frequently stranding on or being retained on beaches. Conversely, some land-based materials disposed on beaches may not ultimately end up in the marine environment, and thus available to turtles. However, despite these constraints, beach debris has widely been used as an indicator of marine debris, for several key reasons [59], [60]. First, it is much less resource intensive to monitor beach debris, and collected debris can be characterized comprehensively, unlike with visual at-sea sampling. Second, because debris accumulates on beaches, statistically robust sample sizes can be gathered, while in-water sampling can lead to a paucity of data and the need to extrapolate from small sample sizes [60]. Finally, items on the beach are in dynamic flux with the nearshore marine environment, and can easily become resuspended [61], so while not ideal, beach debris measurements provide a reasonable proxy for environmental availability. However, it is recommended that more in-water sampling of marine debris be carried out to provide quantitative estimates of marine debris and types of marine debris, especially in areas where turtles are likely to occur.

Research in Australia and elsewhere has shown an inverse correlation between the amount of beach debris and the distance from major population centers [3], [62], suggesting that neritic turtles in SE Queensland, near Australia’s 3^rd^ largest city, Brisbane (population >2 million), might come into contact with different amounts of debris than would open ocean turtles. Despite this, pelagic turtles in this study are more likely to ingest debris than are the benthic turtles. This leads us to speculate as to whether pelagic turtles encounter increased amounts of debris in oceanic gyres and in wind rows [63], whether they are less selective due to the decreased food availability in the open ocean, or whether their feeding ecology simply places them at higher risk for debris ingestion.

### Conclusions

This study found that pelagic and neritic turtles exhibit significant differences in their likelihood of ingesting debris, as well as in their selectivity of debris types. These differences are likely related to their life style and feeding habits, but may also be linked to differing debris availability in the habitats that they frequent. In order to assess population scale impacts from debris ingestion, a greater understanding of the distribution of debris, as well as the long and short-term impacts of ingested debris is required. Further research and modeling of debris in both the nearshore and oceanic environment, in addition to research on the lethal and sublethal impacts of various types of debris loading will provide more accurate and precise estimates of what is available to marine wildlife, the likelihood of encounter rate, and ultimately the risks associated with anthropogenic marine debris ingestion.

It is also important to continue conducting necropsies and to create standardized reporting mechanisms, as the percent and types of debris ingested may be used as an indicator of the impacts of marine debris to wildlife, and only with long-term consistent data collection and recording can we begin to understand how this may change through time.

Close to ninety percent of the debris ingested by turtles in this study was plastic in origin. Observationally it would appear over half of the animals had a non-trivial debris load. As the global production and use of plastics continues to rise, it is likely that impacts to turtles will not abate. Additionally, the observed trend towards selectivity for rubber items, particularly balloons, highlights the need for targeted pollution prevention plans. Appropriate waste disposal measures to reduce debris through local measures would help to decrease the amount of anthropogenic debris entering the ocean; an important first step in reducing encounter rates and impacts to marine wildlife from ingestion or entanglement.
