# Correction: To Eat or Not to Eat? Debris Selectivity by Marine Turtles

**DOI:** 10.1371/annotation/0215f07d-0265-485c-966f-aee192a18313

**Published:** 2012-10-15

**Authors:** Qamar Schuyler, Britta Denise Hardesty, Chris Wilcox, Kathy Townsend

There were errors in the placement of the figure legends. The legend published with Figure 3 should be with Figure 5. The legend published with Figure 4 should be with Figure 3. The legend published with Figure 5 should be with Figure 4. 

