# Correction: To Eat or Not to Eat? Debris Selectivity by Marine Turtles

**DOI:** 10.1371/journal.pone.0221846

**Published:** 2019-08-26

**Authors:** Qamar Schuyler, Britta Denise Hardesty, Chris Wilcox, Kathy Townsend

The following information is missing from the Competing Interest statement: BDH and CW received funding from the Shell Social Investment Fund. This funding began in 2011, after completion of this study, and did not support the work reported in this article.

Additionally, the authors are providing the available individual-level data underlying the results as Supporting Information file S1 Dataset. The individual-level data relating to more specific subcategories of debris type are no longer available.

## Supporting information

S1 Dataset Debris dataset from beach surveys and turtle necropsies. (CSV)
